# The effects of sex hormones on the size of intestinal lipoproteins

**DOI:** 10.3389/fphys.2023.1316982

**Published:** 2023-12-19

**Authors:** Andromeda M. Nauli, Ann Phan, Patrick Tso, Surya M. Nauli

**Affiliations:** ^1^ Department of Biomedical Sciences, Western Michigan University Homer Stryker M.D. School of Medicine, Kalamazoo, MI, United States; ^2^ Desert Valley Hospital, Victorville, CA, United States; ^3^ Department of Pathology and Laboratory Medicine, University of Cincinnati College of Medicine, Cincinnati, OH, United States; ^4^ Department of Biomedical and Pharmaceutical Sciences, Chapman University, Irvine, CA, United States; ^5^ Department of Medicine, University of California, Irvine, Irvine, CA, United States

**Keywords:** digestion, fat, gender, chylomicron, gut, absorption, VLDL, testosterone

## Abstract

Larger intestinal lipoproteins are more likely to be retained longer in the intestinal wall, allowing more time for their fat to be hydrolyzed and subsequently taken up by the abdominal viscera. Since men generally accumulate more abdominal visceral fat than women, we sought to determine if males produce larger intestinal lipoproteins compared to females. Using the conscious lymph fistula mouse model, we discovered that the male mice indeed produced larger intestinal lipoproteins than the female mice when they were intraduodenally infused with lipid emulsion. We then employed our differentiated Caco-2 cell model with semipermeable membrane system to determine the effects of sex hormones on the size of intestinal lipoproteins. Lipoprotein size was quantitatively measured by calculating the ratio of triglycerides (TG)/Apolipoprotein B (ApoB) and by analyzing their transmission electron micrographs. Our studies showed that while there was no dose-dependent effect of estrogen and progesterone, testosterone significantly increased the size of lipoproteins. When these hormones were combined to resemble the physiological concentrations observed in males and the different ovarian cycle phases in premenopausal females, both the male and luteal groups had significantly larger lipoproteins than the ovulatory group; and the male group also had significantly larger lipoproteins than the follicular group. The ovulatory group secreted a significantly lower amount of TG than the male and luteal groups. ApoB was comparable among all these groups. These findings support our hypothesis that, through their testosterone effects, males are more likely to produce larger intestinal lipoproteins. Larger lipoproteins tend to remain longer in the intestinal wall and may facilitate fat uptake preferentially by the abdominal viscera. Our studies may partly explain why men are more prone to accumulating abdominal visceral fat, which is an independent predictor of mortality.

## 1 Introduction

It is evident that lipid metabolism, cardiovascular risk factors, and body fat distribution differ between men and premenopausal women (Meloni et al., 2023). Compared to men, premenopausal women have a more favorable lipid profile, characterized by lower total cholesterol, lower low-density lipoproteins (LDL), lower TG, and higher high-density lipoproteins (HDL) (Wang et al., 2011). Although LDL constitutes an important cardiovascular risk factor, its significance appears to be greater in men than in women. Conversely, plasma TG appears to be a significant risk factor in women compared to men (Regitz-Zagrosek et al., 2007).

One of the most apparent sex differences lies in the distribution of body fat, with women predominantly storing fat in the subcutaneous depot and men in the abdominal visceral depot (Grauer et al., 1984). Importantly, the accumulation of abdominal visceral fat is key criterion for diagnosing metabolic syndrome (Grundy et al., 2005). Our previous hypothesis proposed that these sex-based disparities in body fat accumulation are partially attributed to differences in dietary fat absorption (Nauli and Matin, 2019). Specifically, larger intestinal lipoproteins tend to remain longer in the intestinal wall (Takahara et al., 2013), allowing more time for the hydrolysis of their fat content and uptake by the abdominal viscera (Harvey et al., 2005; Escobedo et al., 2016). Since men generally accumulate more abdominal visceral fat than women (Grauer et al., 1984), our study aims to ascertain whether males produce larger intestinal lipoproteins than females. Importantly, studies have also shown that men stored more of their ingested fat in the abdominal viscera than women (Marin et al., 1996; Votruba et al., 2007).

Even though men and women had comparable fat intake as percent of total energy (33.6% and 33.5%, respectively), men consumed more total calories (2,504 kcals) than women (1,771 kcals) (Wright and Wang, 2010). Consequently, men consume more total dietary fat than women. As intestinal cells produce larger lipoproteins when challenged with dietary lipid (Nauli et al., 2006; Nauli et al., 2014), this factor alone can explain why men are more prone to producing larger intestinal lipoproteins. The notion that sex hormones can impact intestinal lipid absorption was initially proposed in the late 1970s by Vahouny et al. (1977), Vahouny et al. (1980). Their studies revealed that female rats exhibited higher VLDL protein production compared to that of male rats, even when both sexes were subjected to the same amount of dietary fat. These studies implied that females predominantly utilize VLDL as their primary gut lipoprotein. Notably, both VLDLs and chylomicrons are both the predominant intestinal lipoproteins, with VLDLs being smaller than chylomicrons.

However, the average size of intestinal lipoproteins between males and females has not been directly compared to date. The primary goal of our current studies is to address this gap. Additionally, we aim to investigate the effect of sex hormones on the size of intestinal lipoproteins.

## 2 Materials and methods

### 2.1 Animals

Five female and four male C57BL/6 mice were used. All mice were between 4- and 9-month-old. The animal study was reviewed and approved by the Institutional Animal Care and Use Committee at the University of Cincinnati.

#### 2.1.1 Lymph and duodenal cannulation

The intestinal lymph ducts of the anesthetized mice (ketamine, 80 mg/kg and xylazine, 20 mg/kg) were cannulated with polyvinyl chloride tubing as previously described by Bollman et al. (1948). However, we made the following alterations: the suture of the lymph cannula was replaced by cyanoacrylate glue (Krazy Glue, Itasca, IL, United States) and the tubing was inserted into the duodenum through a fundal incision of the stomach. After the surgery, the mice were intraduodenally infused overnight with 5% glucose in saline at a rate of 0.3 mL/h. The 5% glucose in saline solution was replaced with the prepared lipid infusate (see below) the next morning.

#### 2.1.2 Lipid infusion and lymph collection

Triolein (TG), cholesterol, and egg phosphatidylcholine were dissolved in chloroform. The chloroform-lipid mixture was subjected to a gentle stream of nitrogen gas, and the chloroform-free lipid mixture was then emulsified with sodium taurocholate in phosphate-buffered saline (pH 6.4) and sonicated until the solution appeared homogenous. The lipid emulsion was intraduodenally infused at a constant rate of 0.3 mL per hour into the mice for 6 h. The hourly infusate contained 4 μmol triolein, 0.78 μmol cholesterol, 0.78 μmol phosphatidylcholine, and 5.7 μmol sodium taurocholate in phosphate-buffered saline (Nauli et al., 2006). Lymph samples were collected for 1 hour before lipid infusion (fasting lymph) and for another hour during the 6^th^ hour of lipid infusion (6^th^ hour lymph). These two time points were selected because they represent the fasting and postprandial lymph, respectively. Based on our extensive experience in lymph fistula mouse model (Nauli et al., 2006; Liu et al., 2021), the lymphatic TG transport has usually reached the steady state by the 6^th^ hour of lipid infusion. Only negligible quantity of infused TGs remains in the lumen of the small intestine and colon at the end of our lipid infusion, indicating that almost all of our infused TGs are hydrolyzed and taken up by the enterocytes.

At the end of the 6-h lipid infusion, the estrous cycle of the female animals was determined by vaginal smear/cytology. Afterwards, all the animals were euthanized.

#### 2.1.3 Imaging analysis of the size of intestinal lipoproteins from female and male mice

Both the fasting (not diluted) and 6^th^ hour lymph samples (diluted 1:4 (v/v) with sterile water) were overlaid with carbon-coated formvar film on a 400-mesh copper grid. After drying them with filter paper, the grids were added with 2% phosphotungstic acid (pH 6.0) and redried with filter paper. The samples were then subsequently examined by using a transmission electron microscope (JEOL JEM-1230), and the representative images were captured (Nauli and Whittimore, 2015). The size of the lipoprotein particles (approximately 800 particles per lymph sample) was measured by using Adobe Photoshop software. Unfortunately, analyzing and capturing transmission electron micrographs of intestinal lipoproteins were cost prohibitive, allowing us to only have limited number of samples.

### 2.2 Caco-2 cells

Caco-2 (Cancer Coli-2) cells, which were derived from a 72-year-old Caucasian male suffering from colorectal adenocarcinoma (Fogh et al., 1977), have been characterized both biochemically and microscopically for their ability to produce intestinal lipoproteins (Nauli et al., 2014; Nauli and Whittimore, 2015). Other cells lines, including HT-29 cells derived from a female individual, have not been shown to produce intestinal lipoproteins effectively. As such, we were only able to use Caco-2 cells in these experiments. These cells were purchased from the American Type Culture Collection and were grown at 37°C supplemented with 5% CO_2_. The growth media consisted of 15% fetal bovine serum in high glucose Dulbecco’s Modified Eagle Medium (DMEM). The experiments were conducted using 13-day post-confluent cells grown on the semipermeable membrane system (6-well plates with polycarbonate membrane inserts, 4.67 cm^2^ growth area per insert, 1 μm pore).

#### 2.2.1 Co-treatment of Caco-2 cells with lipid mixture and sex hormones

To study the effects of sex hormones on intestinal lipoprotein secretion, prewashed Caco-2 cells were incubated for 4 h with 1.0 mL of prefiltered lipid mixture (2.0 mM oleic acid, 1.36 mM egg-phosphatidylcholine, and 1.0 mM sodium taurocholate in the growth media) in the apical compartment and 2.0 mL of sex hormone-supplemented growth media in the basolateral compartment. All sex hormones were dissolved in dimethyl sulfoxide (DMSO) to achieve their 1,000x concentrations and were then freshly diluted with the growth media to their desired 1x concentrations. As such, DMSO served as our vehicle control and all groups received the same amount of DMSO. The condition used in these experiments (13-day post-confluence, 2.0 mM oleic acid, 1.36 mM egg-phosphatidylcholine, and the 4-h incubation time) has previously been determined to be the most optimal in producing lipoproteins (Nauli et al., 2014; Nauli and Whittimore, 2015).

For the estrogen studies, the following estradiol concentrations were used: 0, 50, 100, and 200 pg/mL. For progesterone: 0, 0.5, 1, and 10 ng/mL. For testosterone: 0, 3, 6, and 9 ng/mL. To better resemble the physiological condition, we also studied the effects of the combination of these 3 hormones according to their reported concentrations in young normal-weight men and women during follicular, ovulatory, and luteal phase (Sherman and Korenman, 1975) (see Table 1).

**TABLE 1:** The combinations of sex hormones that represent men and different phases of ovarian cycle. Caco-2 cells were incubated with different mixtures of estrogen, progesterone, and testosterone to resemble the physiological concentrations in men and different phases of ovarian cycle.

		Estrogen (pg/mL)	Progesterone (ng/mL)	Testosterone (ng/mL)
Female	Follicular	50	0.1	0.3
	Ovulatory	200	1.0	0.3
	Luteal	150	10	0.3
Male		20	0.1	6.0

#### 2.2.2 Biochemical analysis of the lipoprotein size

At the end of the 4-h incubation, basolateral media were collected and subjected to sodium chloride density gradient ultracentrifugation. Briefly, the collected samples were added with sodium chloride and distilled water to bring their density to 1.2 g/mL and volume to 8.0 mL. The 1.2 g/mL density samples were then gently overlaid with 0.5 mL water (density = 1.0 g/mL) and were spun at 65,000 rpm (429,782 × g) for 24 h at 4°C by using the ultracentrifuge T-1270 rotor. The top 0.5 mL lipoprotein fractions were immediately isolated and analyzed.

The isolated fractions were biochemically analyzed for their TG using the Wako L-type Triglyceride M kit and for their ApoB using our previously described enzyme-linked immunosorbent assay (ELISA) (Nauli et al., 2014). Since there is only one molecule of ApoB per lipoprotein particle (Albers et al., 1996), the relative size of intestinal lipoproteins can be indirectly determined by their TG/ApoB ratio.

#### 2.2.3 Imaging analysis of the size of lipoproteins from sex hormone-treated Caco-2 cells

After the fractions were overlaid with carbon-coated formvar film on a 400-mesh copper grid and negatively stained with 2% phosphotungstic acid (pH 6.0), the size of the lipoprotein particles (approximately 200 particles) from the follicular, ovulatory, luteal, and male groups (*n* = 3 each) were examined using a transmission electron microscope (JEOL 2800). The representative images were captured and analyzed as described above.

### 2.3 Statistical analysis

**FIGURE 1:**
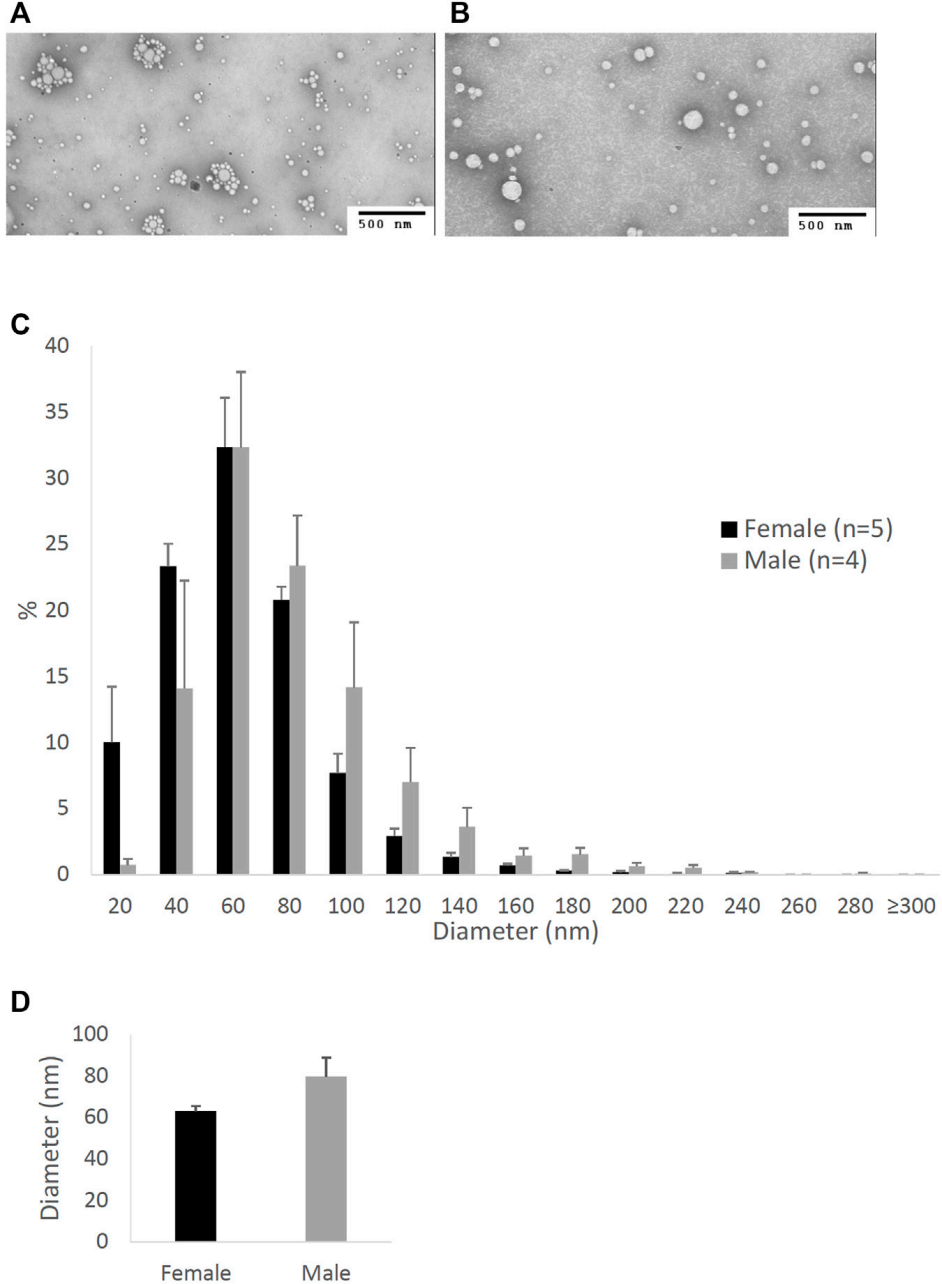
Particle size of lipoproteins from the lymph of mice that were intraduodenally infused with glucose/saline solution. Female (*n* = 5) and male (*n* = 4) mice were intraduodenally infused with glucose/saline solution and their intestinal lymph was collected for 1 hour. The lipoproteins in the collected lymph were then analyzed by a transmission electron microscope. The representative lipoprotein micrographs of the female **(A)** and male **(B)** mice, their lipoprotein size distribution **(C)**, and their lipoprotein average size **(D)** are depicted. Scale bars are 500 nm. Values are means ± standard errors.

**FIGURE 2:**
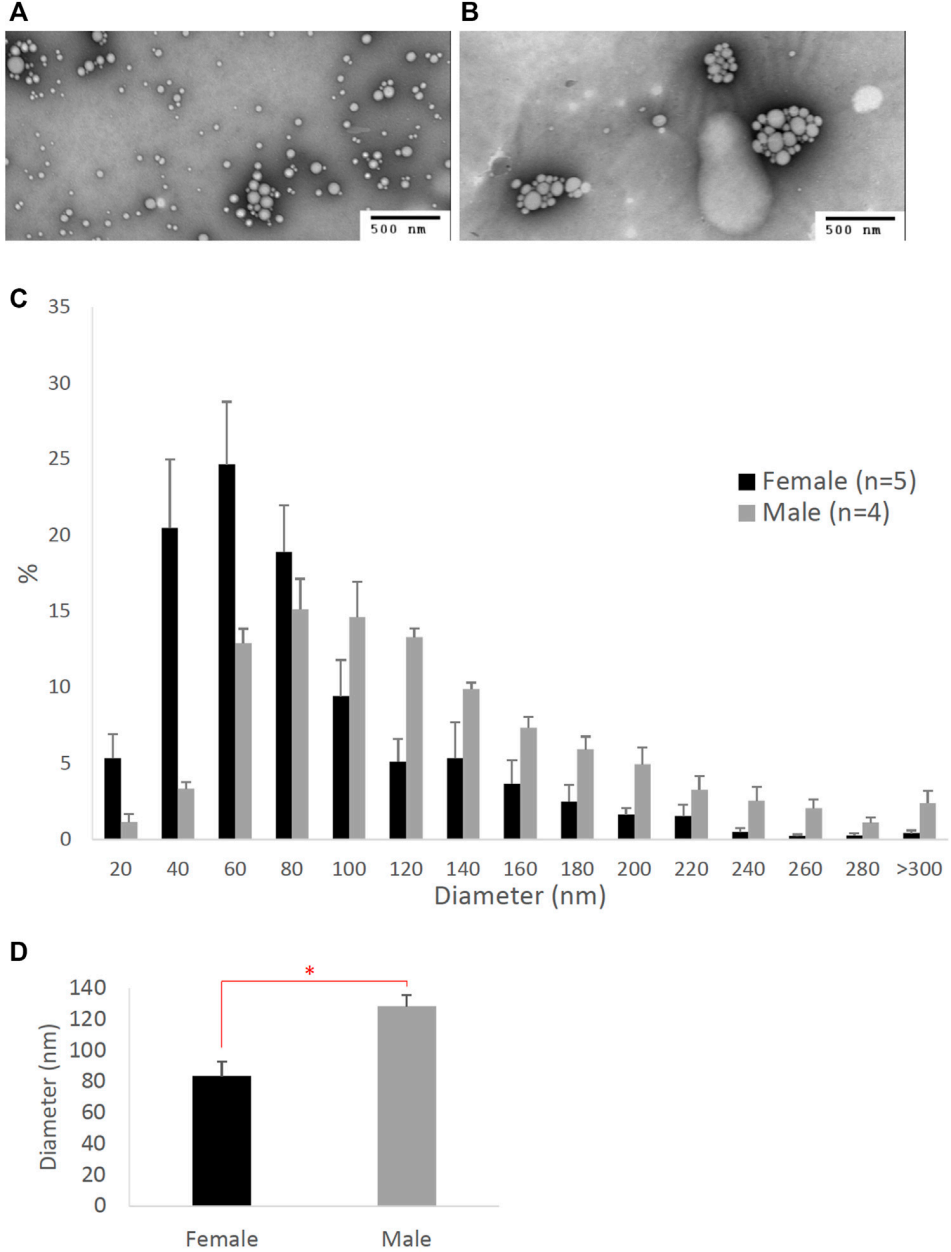
Particle size of lipoproteins from the lymph of mice that were intraduodenally infused with lipid emulsion. Female (*n* = 5) and male (*n* = 4) mice were intraduodenally infused with lipid emulsion for 6 h and their lipoproteins from the intestinal lymph collected at the 6^th^ hour were analyzed by a transmission electron microscope. The representative lipoprotein micrographs of the female **(A)** and male **(B)** mice, their lipoprotein size distribution **(C)**, and their lipoprotein average size **(D)** are depicted. Scale bars are 500 nm. Values are means ± standard errors. The asterisk sign indicates that *p* < 0.05 (two-tailed *t*-test).

**FIGURE 3:**
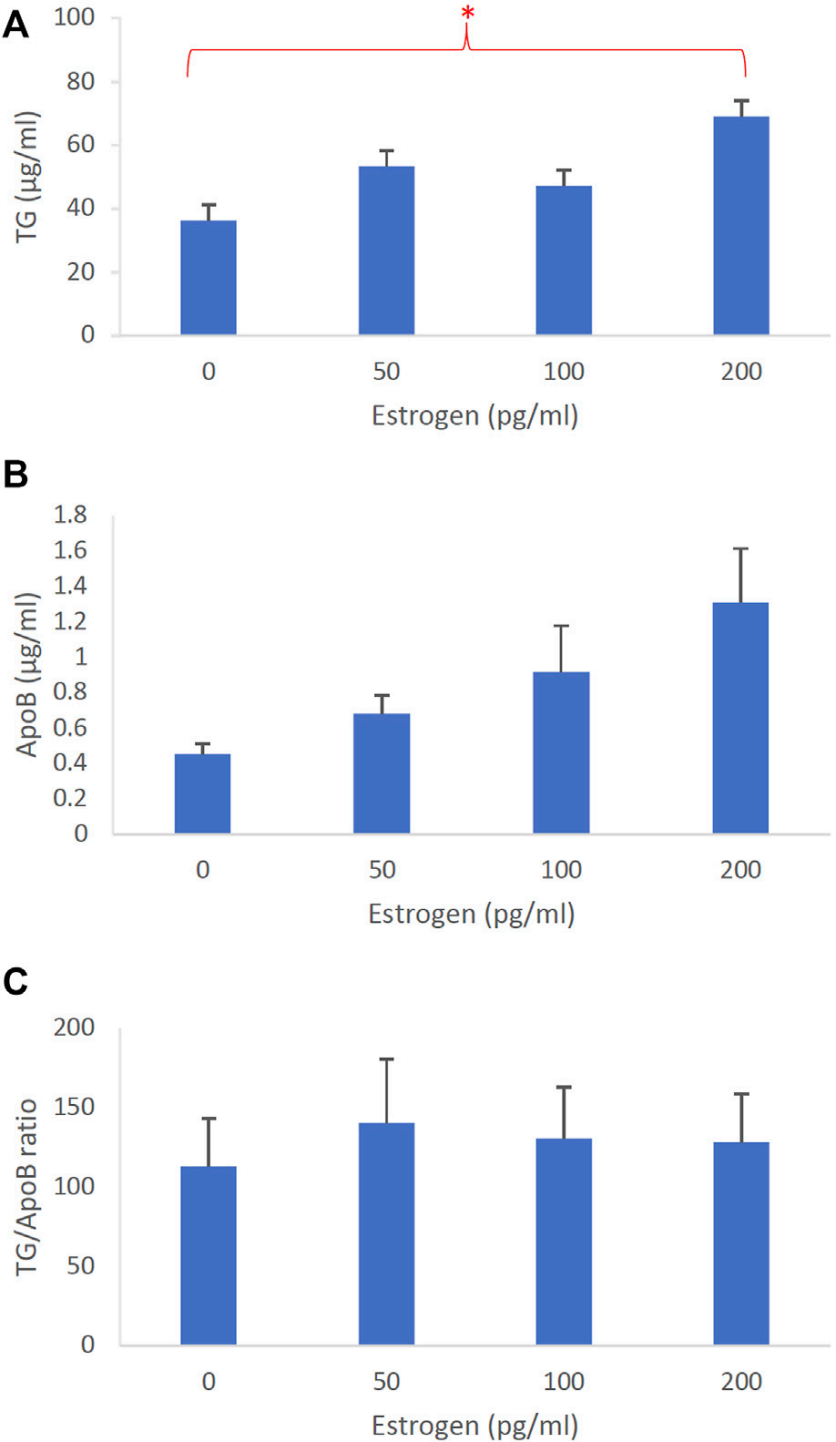
The dose-dependent effects of estrogen on intestinal lipoproteins. Using a semipermeable membrane system, the differentiated Caco-2 cells (*n* ≥ 14) were incubated for 4 h with lipid mixture in their apical compartment and 0, 50, 100, or 200 pg/mL of estrogen in their basolateral compartment. The lipoproteins were then isolated from their basolateral media by using NaCl gradient ultracentrifugation. The lipoprotein TG **(A)**, ApoB **(B)**, and TG/ApoB ratios **(C)** are depicted. The TG/ApoB ratios represent the sizes of lipoproteins. Values are means ± standard errors. The asterisk sign indicates that *p* < 0.05 (one-way ANOVA).

**FIGURE 4:**
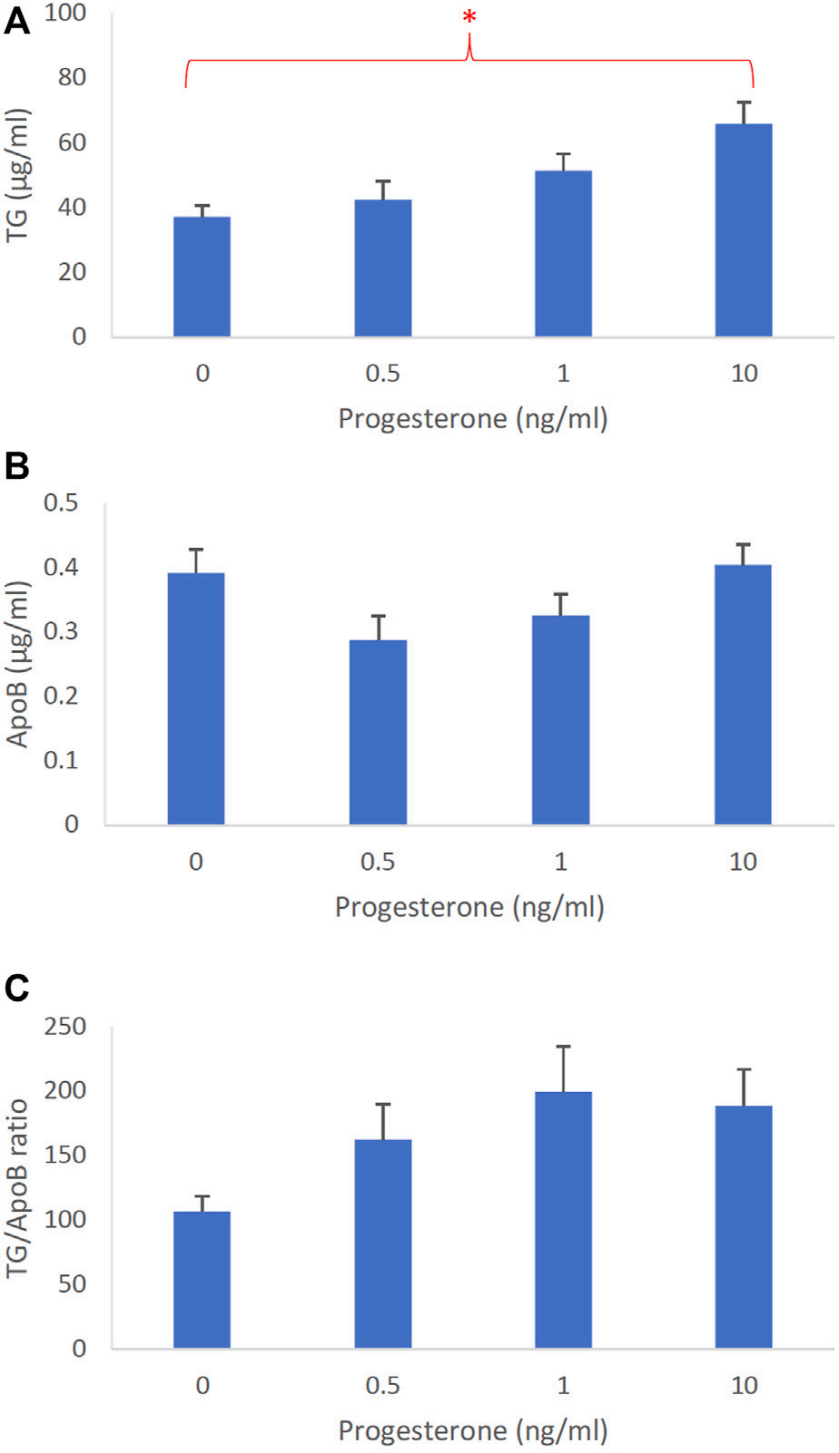
The dose-dependent effects of progesterone on intestinal lipoproteins. Using a semipermeable membrane system, the differentiated Caco-2 cells (*n* ≥ 14) were incubated for 4 h with lipid mixture in their apical compartment and 0, 0.5, 1, or 10 ng/mL of progesterone in their basolateral compartment. The lipoproteins were then isolated from their basolateral media by using NaCl gradient ultracentrifugation. The lipoprotein TG **(A)**, ApoB **(B)**, and TG/ApoB ratios **(C)** are depicted. The TG/ApoB ratios represent the sizes of lipoproteins. Values are means ± standard errors. The asterisk sign indicates that *p* < 0.05 (one-way ANOVA).

**FIGURE 5:**
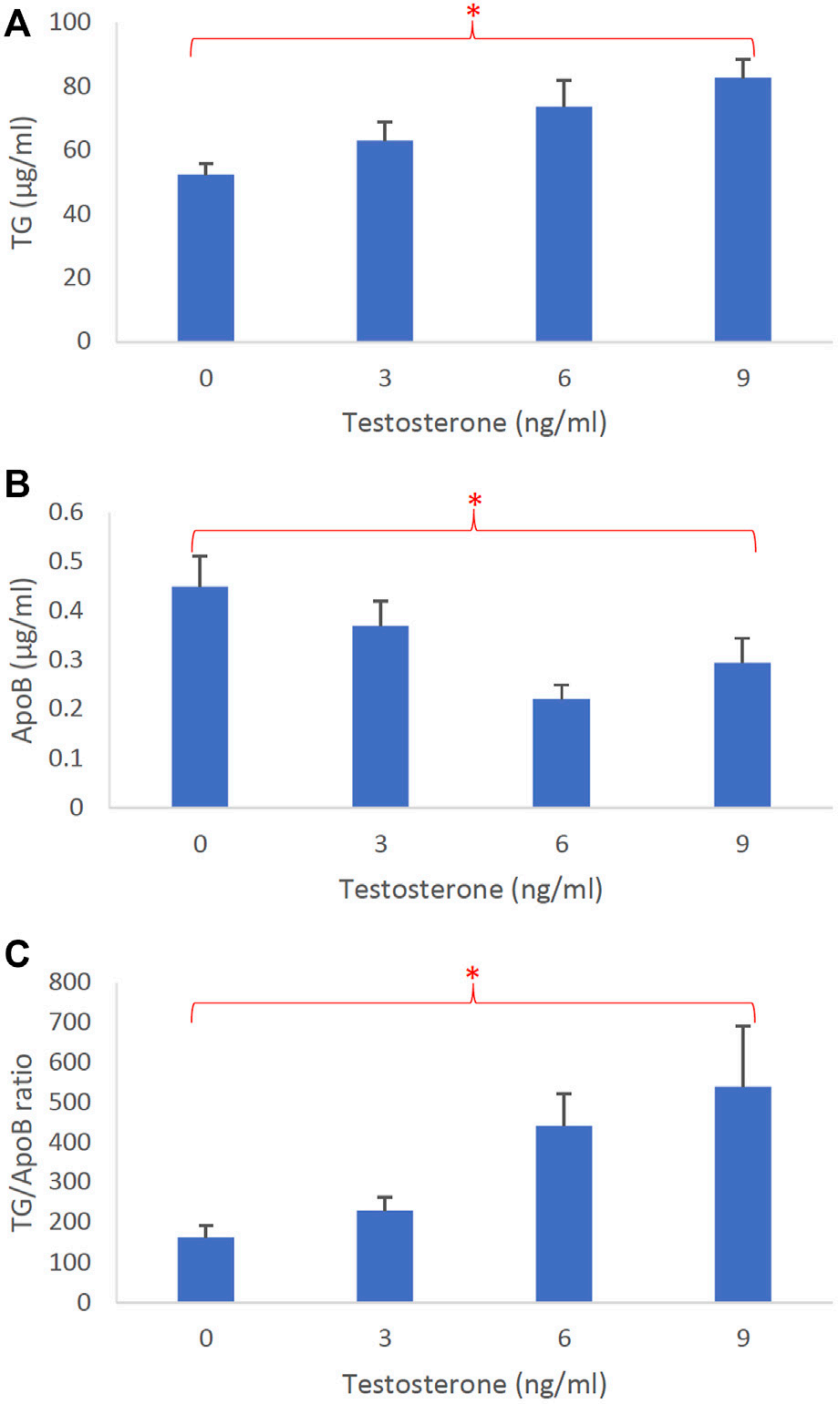
The dose-dependent effects of testosterone on intestinal lipoproteins. Using a semipermeable membrane system, the differentiated Caco-2 cells (n ≥ 14) were incubated for 4 h with lipid mixture in their apical compartment and 0, 3, 6, or 9 ng/mL of testosterone in their basolateral compartment. The lipoproteins were then isolated from their basolateral media by using NaCl gradient ultracentrifugation. The lipoprotein TG **(A)**, ApoB **(B)**, and TG/ApoB ratios **(C)** are depicted. The TG/ApoB ratios represent the sizes of lipoproteins. Values are means ± standard errors. The asterisk signs indicate that *p* < 0.05 (one-way ANOVA).

**FIGURE 6:**
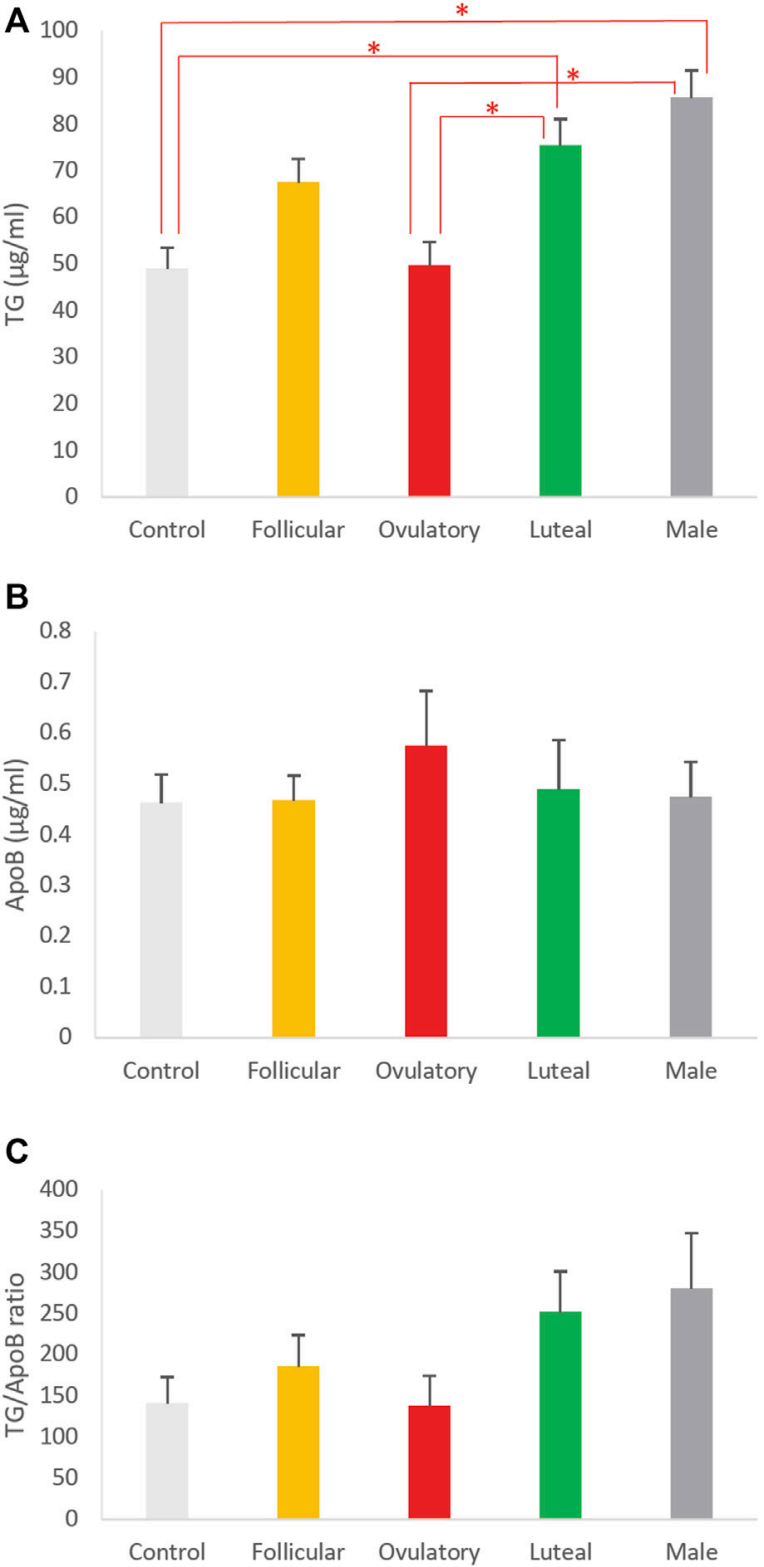
The effects of ovarian cycle on intestinal lipoproteins. Using a semipermeable membrane system, the differentiated Caco-2 cells (*n* ≥ 12) were incubated with lipid mixture in their apical compartment and sex hormone mixture or vehicle (control) in their basolateral compartment. The concentrations of the sex hormones used resembled those in men and different phases of the ovarian cycle (See Table 1). After 4 h of incubation, the lipoproteins were isolated from the basolateral media by using NaCl gradient ultracentrifugation. The lipoprotein TG **(A)**, ApoB **(B)**, and TG/ApoB ratios **(C)** are depicted. The TG/ApoB ratios represent the sizes of lipoproteins. Values are means ± standard errors. The asterisk signs indicate that *p* < 0.05 (one-way ANOVA; Tukey *post hoc* tests were only performed when one-way ANOVA showed statistical significance).

**FIGURE 7:**
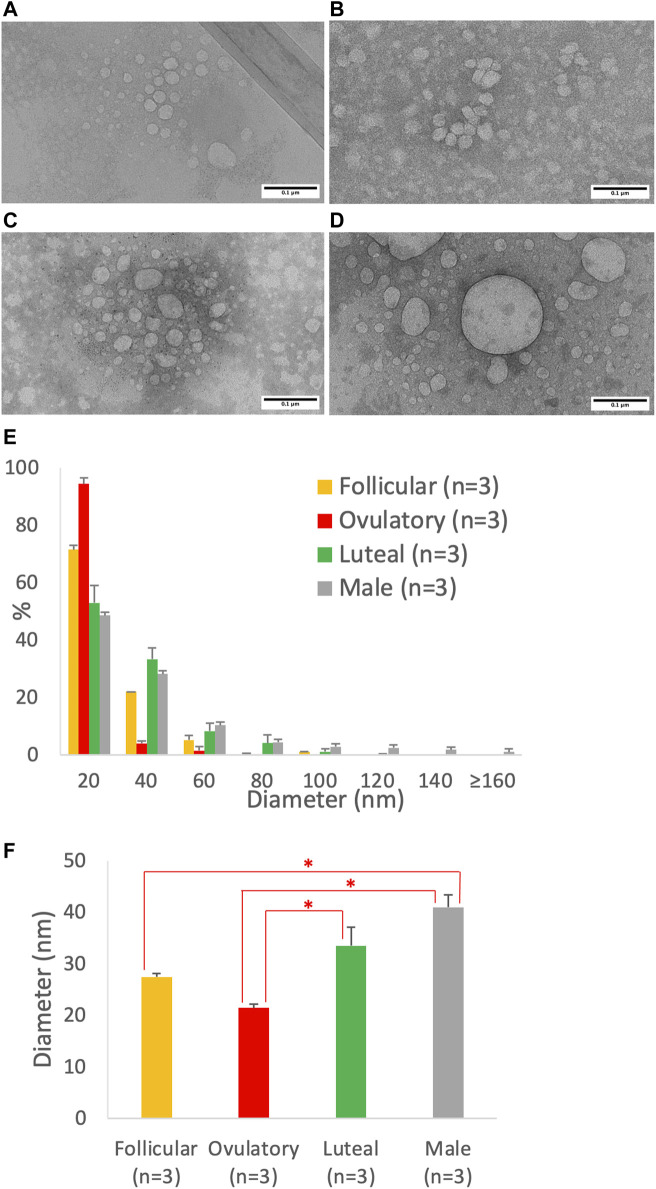
Particle size of lipoproteins from Caco-2 cells treated with the combination of sex hormones that resemble men and different phases of ovarian cycle. Using a semipermeable membrane system, the differentiated Caco-2 cells were incubated with lipid mixture in their apical compartment and sex hormone mixture in their basolateral compartment. The concentrations of the sex hormones used resembled those in men and different phases of the ovarian cycle (See Table 1). After 4 h of incubation, the lipoproteins were isolated from the basolateral media by using NaCl gradient ultracentrifugation, and then analyzed by a transmission electron microscope. The representative lipoprotein micrographs of the follicular phase **(A)**, ovulatory phase **(B)**, luteal phase **(C)**, and male **(D)** are depicted. Lipoprotein size distribution **(E)** and average size **(D)** are also presented. Scale bars are 0.1 μm (100 nm). Values are means ± standard errors. The asterisk signs indicate that *p* < 0.05 (one-way ANOVA followed by Tukey *post hoc* tests).

The data shown represent the mean ± standard errors (SE). One-way analysis of variance (ANOVA) was used to determine the statistical significance of three groups or more (Figures 3A–C, Figures 4A–C, Figures 5A–C, Figures 6A–C, Figures 7F). Tukey *post hoc* tests were performed to determine which of the groups were significantly different from one another (Figures 6A, 7F). The two-tailed *t*-test was used for comparison between two groups (Figures 1D, 2D). Statistical analysis was considered significant if *p* < 0.05. All experiments had n ≥ 3 (n represents the total number of inserts for the Caco-2 experiments). To ensure scientific rigor, the Caco-2 experiments were conducted on at least 3 different occasions, i.e., performed on at least 3 different dates.

## 3 Results

### 3.1 Lipoprotein size differences between male and female mice during glucose/saline infusion

Figure 1 compares the size of intestinal lipoproteins from the lymph of the male and female mice intraduodenally infused with 5% glucose in saline solution. These fasting lymph samples were collected for 1 hour immediately before lipid emulsion was infused. The representative micrographs of fasting lipoproteins from the female (Figure 1A) and male mice (Figure 1B) are shown. Their lipoprotein size distribution (Figure 1C) shows that female mice (*n* = 5) produced more lipoproteins with diameters between 20 and 60 nm, whereas male mice (*n* = 4) produced more lipoproteins larger than 60 nm in diameter. The average diameter of the intestinal lipoproteins of the female mice (63.16 ± 2.46 nm) was not statistically different (*p*-value = 0.094) from that of the male mice (79.74 ± 9.25 nm) (Figure 1D). Of the 5 female mice, 3 were in estrus, 1 in proestrus, and 1 in diestrus.

### 3.2 Lipoprotein size differences between male and female mice during lipid infusion

Figure 2 compares the size of intestinal lipoproteins from the 6^th^-hour lymph of the male and female mice intraduodenally infused with lipid emulsion. As shown, female mice produced smaller intestinal lipoproteins (Figure 2A) than male mice (Figure 2B). Their lipoprotein size distribution (Figure 2C) shows that female mice (*n* = 5) produced more lipoproteins with diameters between 20 and 80 nm, whereas male mice (*n* = 4) produced more lipoproteins larger than 80 nm in diameter. As displayed in Figure 2D, the average diameter of the intestinal lipoproteins of the female mice (83.35 ± 9.35 nm) was significantly smaller (*p*-value = 0.0083) than that of the male mice (128.41 ± 7.28 nm).

### 3.3 The dose-dependent effects of sex hormones on TG, ApoB, and TG/ApoB ratio

To determine the effects of sex hormones on the size of intestinal lipoproteins, we utilized our Caco-2 cell model. Figure 3 shows the dose-dependent effects of estrogen on intestinal lipoprotein TG (Figure 3A), ApoB (Figure 3B), and TG/ApoB ratio (Figure 3C) (*n* ≥ 14). Estrogen significantly (*p* = 0.013) increased intestinal lipoprotein TG. However, there was a tendency for estrogen to also increase intestinal lipoprotein ApoB (*p* = 0.062). Consequently, the TG/ApoB ratio did not appear to be affected by estrogen (*p* = 0.96), indicating that estrogen did not alter the size of the intestinal lipoproteins. Collectively, these data suggest that estrogen tended to increase the number of lipoprotein particles but did not affect their size (“more of the same size”).

The dose-dependent effects of progesterone on intestinal lipoprotein TG, ApoB, and TG/ApoB ratio (*n* ≥ 14) are shown in Figures 4A–C, respectively. Progesterone significantly (*p* = 0.0026) increased intestinal lipoprotein TG but did not appear to significantly (*p* = 0.064) affect the ApoB secretion. Since progesterone gradually increased the intestinal lipoprotein TG/ApoB ratios, progesterone was likely capable of increasing−albeit not significantly (*p* = 0.086)−the size of intestinal lipoproteins. These data cooperatively suggest that progesterone tended to increase the size of intestinal lipoproteins by increasing their TG incorporation (“larger but of the same number”).

Figure 5 represents the dose-dependent effects of testosterone on intestinal lipoprotein TG (Figure 5A), ApoB (Figure 5B), and TG/ApoB ratio (Figure 5C) (*n* ≥ 14). Testosterone significantly increased TG (*p* = 0.0046) and TG/ApoB ratio (*p* = 0.0040) but decreased ApoB (*p* = 0.017). These data indicate that testosterone led to the production of “fewer but larger” intestinal lipoproteins.

### 3.4 The effects of ovarian cycle on TG, ApoB, and TG/ApoB ratio

To determine how different phases of ovarian cycle affect the size of intestinal lipoproteins, we examined the combined effects of these 3 hormones based on their concentrations observed in men and women in their follicular, ovulatory, and luteal phase. We also included a control group that consisted of vehicle (DMSO) without any sex hormones. The effects of the ovarian cycle on the intestinal lipoprotein TG, ApoB, and TG/ApoB ratio are shown in Figures 6A–C, respectively. The ovarian cycle had a significant effect on the intestinal lipoprotein TG (*p* = 0.0000092) but not on the ApoB (*p* = 0.87) and TG/ApoB ratio (*p* = 0.14).

Tukey *post hoc* tests were subsequently performed to determine which of the groups were significantly different from one another in their intestinal lipoprotein TG secretion. As shown in Figure 6A, the ovulatory group had significantly lower intestinal lipoprotein TG than the luteal (*p* = 0.0096) and male (*p* = 0.00010). Similarly, the control group had significantly lower intestinal lipoprotein TG than the luteal (*p* = 0.0073) and male (*p* = 0.000070). There were no statistical differences between ovulatory and control (*p* = 0.99), ovulatory and follicular (*p* = 0.14), control and follicular (*p* = 0.11), follicular and luteal (*p* = 0.84), follicular and male (*p* = 0.12), and luteal and male (*p* = 0.65).

We did not perform Tukey *post hoc* tests for the effects of ovarian cycle on ApoB and TG/ApoB ratio because their one-way ANOVA tests did not show any significant difference. Next, we compared the size of the intestinal lipoproteins from the follicular, ovulatory, luteal, and male groups by imaging analysis.

### 3.5 Imaging analysis of the size of lipoproteins from Caco-2 cells treated with different combinations of sex hormones

Using imaging analysis, we compared the size of the intestinal lipoproteins isolated from the follicular (Figure 7A), ovulatory (Figure 7B), luteal (Figure 7C), and male (Figure 7D) groups. The lipoprotein size distribution (Figure 7E) shows that the ovulatory and follicular groups secreted lipoproteins that were mostly 20 nm in diameter. Luteal and male groups were quite similar in their particle size distribution, but male group produced more particles that were larger than 80 nm in diameter. The effect of ovarian cycle on the lipoprotein size was statistically significant (*p* = 0.0014). As shown in Figure 7F, the ovulatory group had smaller lipoproteins than both the luteal (*p* = 0.020) and male (*p* = 0.0011) group. The follicular group also had smaller lipoproteins than the male group (*p* = 0.011). However, there were no statistical differences between ovulatory and follicular (*p* = 0.29), follicular and luteal (*p* = 0.28), and luteal and male (*p* = 0.16).

## 4 Discussion

It is increasingly clear that abdominal visceral fat is detrimental to health not only in men (Kuk et al., 2006) but also in women (Koster et al., 2015). Men, however, are more likely than premenopausal women to develop abdominal visceral fat (Grauer et al., 1984). The reasons for the sex differences in body fat accumulation remain unclear, but we previously proposed that the small intestine plays several essential roles (Nauli and Matin, 2019). The small intestine is the organ that releases the most amount of TG, predominantly in the form of intestinal lipoproteins, namely VLDL and chylomicron. Large intestinal lipoproteins are more likely to be retained in the intestinal wall (Takahara et al., 2013), allowing their TG to be hydrolyzed and taken up by the abdominal viscera. Indeed, studies have shown that the accumulation of large intestinal lipoproteins in the abdominal wall, caused by the leaky lymphatics, was responsible for the significant gain of abdominal visceral fat (Harvey et al., 2005; Escobedo et al., 2016). Therefore, larger intestinal lipoproteins are likely to contribute to the development of the abdominal visceral fat.

To our knowledge, there has not been any direct comparison of the intestinal lipoprotein size between males and females. Our studies here showed that when they were intraduodenally infused with glucose/saline, female mice produced relatively smaller intestinal lipoproteins than male mice, but their difference was not statistically significant (Figure 1). However, this difference became significant when they were intraduodenally infused with lipid emulsion (Figure 2). Note that these mice were all provided with the same overnight intraduodenal infusion of 5% glucose in saline before being challenged with the same amount of lipid emulsion. Therefore, our data imply that the differences in their intestinal lipoprotein size were partly due to the sex hormones. Since the estrous cycle of mice has different sex hormone profiles than the ovarian cycle of humans, we utilized our Caco-2 cell model to further study the effects of sex hormones and ovarian cycle on the size of intestinal lipoproteins.

All sex hormones, including estrogen, progesterone, and testosterone, increased intestinal TG secretion (Figures 3A, 4A, 5A, respectively). However, only testosterone decreased the intestinal ApoB secretion, resulting in fewer intestinal lipoproteins being produced (Figures 3B, 4B, 5B). These results are consistent with our recently published studies that showed ApoB was not affected by the female sex hormones (Liu et al., 2021). It is important to note, however, that our previous mice studies used different concentrations of sex hormones. Therefore, direct comparisons between these studies should be made with caution.

Conceivably, testosterone also increased the size of intestinal lipoproteins, as evidenced by its significant dose-dependent effect on TG/ApoB ratio (Figure 5C). Progesterone also gradually increased the TG/ApoB ratios from around 100 to 200, but the increase was not statistically significant (Figure 4C). Estrogen did not cause a gradual increase in TG/ApoB ratios as their ratios hovered around 100–150 (Figure 3C). From these experiments, we conclude that testosterone was the sex hormone with the strongest capability of increasing the size of intestinal lipoproteins. It led to the production of “significantly fewer but larger” intestinal lipoproteins. In contrast, estrogen appeared to trigger “slightly more of the same size” of intestinal lipoproteins. Progesterone stimulated “slightly larger but of the same number” of intestinal lipoproteins. In this regard, the testosterone and estrogen effects were rather opposite to each other, and the progesterone effects were somewhat in between those of testosterone and estrogen.

To further investigate the effects of ovarian cycle on intestinal lipoproteins, our Caco-2 cells were exposed to a combination of different sex hormone concentrations resembling those seen in the follicular phase, ovulatory phase, luteal phase, and men (Table 1). As shown in Figure 6A, the control group (vehicle without any sex hormones) had the lowest intestinal lipoprotein TG, followed by the ovulatory, follicular, luteal, and male group. There was, however, no significant difference in the ApoB and TG/ApoB ratio (Figures 6B, C, respectively).

We then analyzed the transmission electron micrographs of the intestinal lipoproteins (Figure 7) and found that the ovarian cycle had a significant effect on the size of intestinal lipoproteins. The ovulatory group had significantly smaller intestinal lipoproteins than both the luteal and male group; and the follicular group also had significantly smaller intestinal lipoproteins than the male group. The ranking order obtained from the imaging analysis agree with that obtained from the biochemical analysis. The ovulatory group had the smallest intestinal lipoproteins, followed by follicular, luteal, and male group. The calculated TG/ApoB ratios appeared to have more variance than the data obtained from imaging analysis, which may explain why the ovarian cycle showed a statistically significant effect on lipoprotein size measured from the transmission electron micrographs but not from the calculated TG/ApoB ratios.

Collectively, our data indicated that the combination of sex hormones that resemble those found in men produced larger intestinal lipoproteins than the combinations that resemble ovulatory and follicular phase. The sex difference in the intestinal lipoprotein size could partly be attributed to testosterone that drove the production of “larger but fewer” lipoproteins. Progesterone also increased—albeit not significantly—the size of these lipoproteins, possibly explaining why the luteal phase, which had the highest level of progesterone, produced larger lipoproteins than the ovulatory phase.

Our data also indicated that sex hormones in male promoted intestinal TG transport that was more efficient than those in the ovulatory and follicular phase. Since TG is positioned in the core of lipoproteins, larger lipoproteins can feasibly transport more TG. However, larger intestinal lipoproteins may be subjected to longer retention in the intestinal wall, favoring the accumulation of the abdominal visceral fat.

From the evolutionary perspective, it is arguably more advantageous for men to have an efficient intestinal TG secretion and the ability to store their fat in the abdominal viscera. Importantly, the release of the stored fat from the abdominal viscera can reach the liver—the key metabolic organ—more effectively than from the subcutaneous depot (Nauli and Matin, 2019). It is also perhaps more advantageous for women to store fat in their subcutaneous depot as it provides a better insulation to the whole body. This may serve as a critical factor for their survival, especially considering that women have lower muscle mass. Of note, smaller intestinal lipoproteins are more likely to enter the blood capillaries than the lymphatic capillaries (Mansbach et al., 1991; Mansbach and Dowell, 1993). Since the blood circulation has a higher pressure than the lymphatic circulation, the portal transport is associated with less retention of intestinal lipoproteins in the gut, which consequently leads to a more robust dietary fat distribution to the subcutaneous depot.

Our current studies are in general agreement with the previous studies. As indicated above, earlier studies showed that female rats had higher VLDL protein production than male rats (Vahouny et al., 1977; Vahouny et al., 1980). Furthermore, our recently published studies showed that female mice had lower lymphatic TG secretion than male mice, which could partly be due to the estrogen-mediated enhancement of vascular endothelial growth factor A (VEGF-A) signaling (Liu et al., 2021). Enhanced VEGF-A signaling has been shown to prevent the movement of intestinal lipoproteins from the lamina propria of the intestinal villi into their lymphatics (Zhang et al., 2018). In addition, it may facilitate the entrance of smaller intestinal lipoproteins into the lumen of blood capillaries.

It is noteworthy to acknowledge the limitation of our studies. Since Caco-2 cells were originally isolated from a male individual, the sex hormone effects in our studies may be different from studies utilizing cells from a female individual, such as HT-29 cells. Sex hormones may interact with sex chromosomes, resulting in potential differences of sex hormone effects in female- and male-derived cells. Additionally, organizational effects of gonadal hormones, which are permanent, may contribute to sex differences in intestinal lipoprotein size (Blencowe et al., 2022). In our experience, however, HT-29 cells are not effective in secreting lipoproteins. Additionally, primary culture derived from adult intestinal tissues is challenging partly due to the short lifespan (3–5 days) of intestinal epithelial cells (Dutton et al., 2019).

From our studies and those of others, it can be concluded that dietary fat absorption is likely different between men and women. We demonstrated in our current studies that sex hormones that resemble those found in men produced larger intestinal lipoproteins than the sex hormones that resemble those found in the ovulatory and follicular phase. This effect could be explained by the ability of testosterone to induce the production of larger intestinal lipoproteins. Progesterone resembled some of the testosterone effects, possibly explaining why cells in the luteal phase condition produced larger intestinal lipoproteins than cells in the ovulatory phase condition. Since men consume more total dietary fat than women (Wright and Wang, 2010), it is reasonable to speculate that this additional factor can further drive the production of larger intestinal lipoproteins in men. Dietary fat is responsible for the production of larger intestinal lipoproteins (Nauli et al., 2006; Nauli et al., 2014). As such, it will be of great interest to determine the effects of the complex interplay between hormonal fluctuation and dietary fat intake on the size of intestinal lipoproteins. Hormonal changes are not only mediated by ovarian cycle, but also by body fat. As body fat increases, free testosterone level tends to increase in females (Bernasconi et al., 1996) but decrease in males (Fui et al., 2014). It remains to be determined if these changes in free testosterone level can alter the intestinal lipoprotein size.

The effects of the combination of sex hormones that resemble postmenopausal women also warrant further investigation. Postmenopausal women are generally older than premenopausal women. However, studies have shown that postmenopausal women were still more likely to develop metabolic syndrome even when compared with age-matched premenopausal women (Eshtiaghi, Esteghamati, and Nakhjavani, 2010). We have previously postulated that aging closes the gap between men and women in their abdominal visceral fat accumulation (Nauli and Matin, 2019). Aging causes lymphatics to be leakier (Zolla et al., 2015), and consequently promotes lipoprotein retention in the intestinal wall.

The sex differences in abdominal visceral fat accumulation are likely mediated by multiple mechanisms. In men, higher testosterone and higher intake of total fat drive the production of larger intestinal lipoproteins. Large intestinal lipoproteins are more likely to be retained longer in the intestinal wall, promoting abdominal visceral fat accumulation. As aging lymphatics become leakier, the sex differences in the abdominal visceral fat accumulation begin to dwindle. Physical inactivity, which is also associated with aging, further promote leaky lymphatics (Hespe et al., 2016).

## Data Availability

The original contributions presented in the study are included in the article/supplementary material, further inquiries can be directed to the corresponding author.

## Abbreviations

ANOVA, analysis of variance; ApoB, apolipoprotein B; Caco-2, cancer coli-2; DMEM, Dulbecco’s Modified Eagle Medium; DMSO, dimethyl sulfoxide; ELISA, enzyme-linked immunosorbent assay; HDL, high-density lipoprotein; LDL, low-density lipoprotein; SE, standard error; TG, triglyceride; VEGF-A, vascular endothelial growth factor A; VLDL, very low-density lipoprotein.
